# Left Cervical Lymphadenopathy Presentation of Metastatic Colorectal Adenocarcinoma

**DOI:** 10.31486/toj.18.0121

**Published:** 2019

**Authors:** Diana Maslov, James Bragg

**Affiliations:** ^1^ The University of Queensland Faculty of Medicine, Ochsner Clinical School, New Orleans, LA; ^2^ Department of Internal Medicine, Ochsner Clinic Foundation, New Orleans, LA; ^3^ Department of Primary Care, Ochsner Clinic Foundation, New Orleans, LA

**Keywords:** *Adenocarcinoma*, *colorectal neoplasms*, *lymphadenopathy*, *lymphatic metastasis*, *metastasis*

## Abstract

**Background:** Colorectal adenocarcinoma, the third most diagnosed cancer in males and the second most diagnosed in females, commonly presents with changes in bowel habits, rectal bleeding, weight loss, fatigue, and abdominal pain. We report the case of a patient with colorectal cancer who had an unusual initial presentation.

**Case Report:** The patient reported dizziness, and physical examination revealed cervical lymphadenopathy. Fine needle aspiration of the lymph node supported a diagnosis of adenocarcinoma, and colonoscopy found a mass in the hepatic flexure that was biopsied and identified as adenocarcinoma. The patient was treated with 5-fluorouracil, leucovorin, and oxaliplatin (FOLFOX) and bevacizumab. Imaging and physical examination 4 months later confirmed resolution of the mass and lymphadenopathy.

**Conclusion:** The patient is regularly followed by hematology/oncology and his primary care physician. He has shown no evidence of recurrence to date. This case supports the necessity of a complete workup and imaging when any solid lymphadenopathy is present.

## INTRODUCTION

Patients with gastrointestinal cancers commonly present with abdominal pain, weight loss, fatigue, and bloody or tarry stools. On physical examination, the patient may have lymphadenopathy, pallor, or abdominal pain on palpation. The rectal examination may reveal bleeding or a mass. Although the occurrence is rare, gastrointestinal cancer can present in the head and neck region. One case described colorectal cancer that metastasized to the thyroid.^1^ In another case, a patient with sigmoid adenocarcinoma presented with cervical lymphadenopathy.^2^ We present the case of a patient with colorectal carcinoma who had no signs of cancer other than cervical lymphadenopathy.

## CASE REPORT

A 59-year-old male with a history of hypertension, gastroesophageal reflux disease, and benign prostatic hypertrophy presented to his primary care physician with symptoms of dizziness when standing up or exiting the bathtub. He described cramping in his lower extremities and hands after walking or working for long periods of time and revealed that he had a cough and cold and had recently been coughing up “whitish yellow sputum.”

The patient was not orthostatic, and his blood pressure was 120/86 mmHg. He had a 1 cm firm, rubbery, movable left cervical lymph node. The rest of the physical examination was unremarkable. Laboratory workup showed hemoglobin of 8.4 g/dL, hematocrit of 29.3%, and mean corpuscular volume of 70 fL, indicative of microcytic anemia. The patient had an iron level of 33 mcg/dL with total iron binding capacity of 475 mcg/dL, correlating with iron deficiency anemia. Computed tomography (CT) scan of the soft tissue neck, chest, abdomen, and pelvis revealed an enlarged precaval lymph node measuring 2.5 cm in the short axis, several enlarged mesenteric lymph nodes with the most prominent measuring 1.8 cm in the short axis, and a peripancreatic lymph node measuring 2.8 cm in the short axis. The patient also had an enlarged left cervical lymph node measuring 2.5 × 2.8 cm that was compressing the left common jugular vein (Figure). Elevation of the right hemidiaphragm and nonspecific thickening of the supralingual soft tissue were noted.

**Figure.**
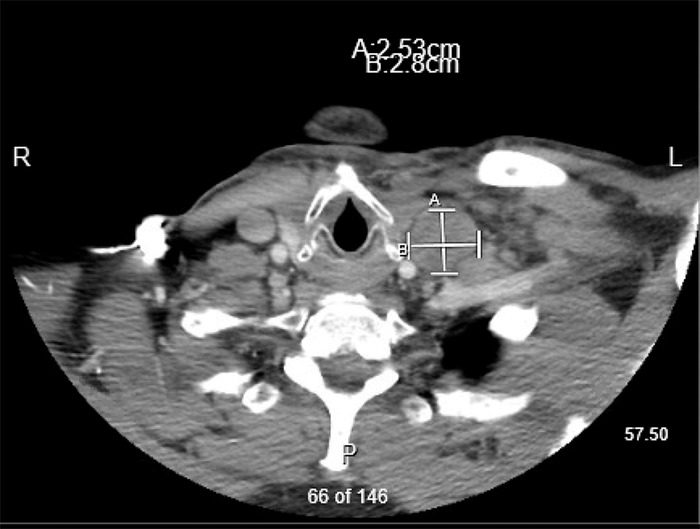
**Computed tomography soft tissue neck scan revealed an enlarged left cervical lymph node measuring 2.5 × 2.8 cm and compressing the left common jugular vein.**

Fine needle aspiration of the left cervical lymph node revealed a metastatic, poorly differentiated carcinoma, supportive of adenocarcinoma. Endoscopy showed normal stomach and duodenum. Colonoscopy revealed a mass in the hepatic flexure in the distal ascending colon that was biopsied and identified as adenocarcinoma. The patient's most recent colonoscopy, performed 8 years prior, showed no significant findings.

The patient was referred to hematology and oncology and was treated with 5-fluorouracil 2,400 mg/m^2^, leucovorin 200 mg/m^2^, and oxaliplatin 85 mg/m^2^ (FOLFOX), along with bevacizumab 5 mg/kg biweekly for 8 months. During the chemotherapy, the patient developed a deep vein thrombosis that was treated with rivaroxaban 20 mg daily for 4 months. He also developed chemotherapy-induced peripheral neuropathy that was treated with gabapentin 600 mg daily for 7 months.

After 59 days of treatment, positron emission tomography scan revealed complete resolution of the lymphadenopathy and almost complete resolution of the colorectal mass. CT scan after 111 days of treatment revealed that the cervical, precaval, mesenteric, and peripancreatic lymph nodes had decreased in size, with no new lymphadenopathy. On physical examination, cervical lymphadenopathy had resolved. The patient is regularly followed by his primary care provider and hematology/oncology team. He remains on maintenance chemotherapy and has shown no evidence of recurrence to date.

## DISCUSSION

Colorectal carcinoma is the third most commonly diagnosed cancer in males and the second most common in females.^3^ In the United States, the incidence and mortality of colorectal cancer have declined as a result of increased screening.^3^ The American Cancer Society recommends that people should start screening when they reach the age of 45 years and continue through the age of 75 years.^3^ Those at increased risk for colorectal cancer should begin screening earlier. At-risk individuals are those with a strong family history of colorectal cancer, a personal history of polyps or inflammatory bowel disease, or a family history of hereditary colorectal cancer syndromes.

The 3 methods most often used to screen for colorectal cancer are a colonoscopy every 10 years, a fecal immunochemical test every year or every 3 years, or a flexible sigmoidoscopy every 5 years.^4^ Our patient had no increased risk of colorectal carcinoma and was being appropriately screened via colonoscopy every 10 years.

Patients with colorectal adenocarcinoma commonly present with changes in bowels and/or bloody stools, anemia, and abdominal pain.^4^ Our patient presented with microcytic and iron deficiency anemia.

Colorectal carcinoma tends to metastasize to the liver, lungs, bone, and/or brain^4^ because of the venous drainage of the intestinal tract via the portal system. Our patient had no liver findings. He had supralingual thickening in the lung, as well as precaval, mesenteric, peripancreatic, and left cervical lymph node enlargement.

Patients with metastatic head and neck cancers often present with cervical lymphadenopathy. Infraclavicular tumors have been known to metastasize to the supraclavicular lymph nodes.^5^ However, patients with metastasis from an infraclavicular cancer presenting with cervical lymphadenopathy are rare.^5^ Gastrointestinal cancers are recognized to metastasize to the left supraclavicular lymph node (Virchow node), known as the Troisier sign.^6^ However, our patient's initial presentation of an enlarged cervical lymph node was unusual.

## CONCLUSION

To our knowledge, this case is the first report of a patient with colorectal adenocarcinoma of the hepatic flexure who presented with cervical lymphadenopathy. In addition to illustrating an unusual initial presentation of colorectal adenocarcinoma, this case highlights the need for a complete patient workup and imaging when any solid lymphadenopathy is present.

## Notes

This article meets the Accreditation Council for Graduate Medical Education and the American Board of Medical Specialties Maintenance of Certification competencies for Patient Care and Medical Knowledge.
